# Removal of Pesticides from Lemon and Vegetables Using Electrolyzed Water Kitchen Devices

**DOI:** 10.3390/molecules29235797

**Published:** 2024-12-08

**Authors:** Waldemar Studziński, Izabela Narloch, Łukasz Dąbrowski

**Affiliations:** Department of Food Analysis and Environmental Protection, Faculty of Chemical Technology and Engineering, Bydgoszcz University of Science and Technology, Seminaryjna 3, 85-326 Bydgoszcz, Poland; lukas@pbs.edu.pl

**Keywords:** electrolyzed water, pesticide degradation, pesticide residue, water treatment

## Abstract

The possibility of using kitchen electrolyzed water devices (EWDs) for removing residual concentrations of pesticides (malathion, fenitrothion, and p,p′-DDT) from lemon, cucumber, and carrot surfaces was tested. Three commercial devices with different parameters were tested, and their effectiveness was compared with traditional washing methods using water. Based on the results, it was found that by using EWDs, the best removal of water-soluble pesticides was achieved with malathion and fenitrothion (reduction of up to 80%). The worst effectiveness was observed for lipophilic DDT, where a reduction of 20 to 40% was noted. Traditional methods proved to be more effective for removing DDT. Our studies have shown that EWDs can effectively remove pesticide residues; however, further studies should be conducted on a wider spectrum of pesticides and the process should be optimized.

## 1. Introduction

It is estimated that 30–40% of food crops are lost due to damage or reduction in their quality from the negative impact of pathogens, insects, weeds, and fungi [1]. Therefore, to ensure the appropriate level of food crop productivity and quality, pesticides must be used [2]. However, excessive or uncontrolled use of pesticides can result in residual concentrations of these compounds remaining in environmental matrices [3].

Due to the dispersion of pesticides in the environment, it is estimated that more than 95% of these compounds can affect non-target organisms. This can threaten living organisms because pesticides are characterized by high durability, are resistant to degradation, and possess toxic effects. In addition, the effects of some pesticides can persist for several decades and negatively affect soil quality [4]. In addition, pesticide residues exhibit the ability to bioaccumulate and biomagnify, leading to the amplification of pesticide concentrations in the food chain [5]. Consuming pesticides in food and water poses a serious health risk [6]. Scientists have shown that the consumption of pesticides is associated with the development of health disorders such as oxidative stress, allergies, growth retardation, mutagenic, carcinogenic, teratogenic, neurotoxic effects, and even death [5,7,8]. The toxicity and effects of each pesticide group are related to the concentration and type of pesticide used. Therefore, pesticide residues are monitored and strictly regulated by government organizations due to their potential toxicity [9]. Each active substance has a maximum residue limit (MRL) that allows national authorities to control whether pesticides have been used correctly [2]. Scientists are researching to develop methods that can be used to remove pesticide residues from different types of vegetables and fruits. Many studies have used traditional methods such as peeling, cutting, washing, rinsing, soaking, blanching, and cooking [10,11,12]. The most effective approach is considered to be peeling or trimming vegetables and fruits to reduce pesticide residues [13]. However, it is difficult to apply these methods to leafy plants, and the skins of vegetables and fruits often contain very valuable nutrients [14]. Recently, treatment in ozonated water has also been used to remove pesticides from food [2], ozone/UV treatment [15], washing with bleach [16], using cold plasma [1], treatment in alkaline water, washing with detergent, treatment in water with sodium bicarbonate, and using ultrasound [17]. However, neither of the methods mentioned guaranteed the complete removal of pesticides from food. The effectiveness of food purification from pesticide residues is influenced by the duration of purification, type of food, type of pesticide, and degree of contamination with pesticide residues [17]. The processes mentioned can effectively remove pesticides from food products, but there is still a gap between scientific or laboratory research and commercial applications. Recently, kitchen electrolyzed water devices (EWDs) have appeared on the market. The devices use the process of water electrolysis, which leads to the production of reactive hydroxyl radicals. As a result of such action, water acquires a negative redox value. The reactions occurring during the treatment process are unstable, so after electrolysis stops, the redox value increases rapidly to its initial value. In addition to the electrolysis process, the devices use ultrasound and have diodes that emit UV radiation. The use of ultrasonic waves in devices enhances the degradation of pesticides from aqueous solution through a mechanism based on pyrolysis inside cavitation bubbles or a mechanism related to the formation of hydroxyl radicals in cavitation bubbles [18]. While UV radiation is one of the most effective factors used to decompose organic compounds, the direct effects of UV radiation may include the formation of new chemical compounds, the breaking of chemical bonds, and, as a result, the destruction of organic substances. Indirect photolysis, on the other hand, takes place under the influence of reactions with •OH radicals, the number and reactivity of which depend on the intensity of ultraviolet radiation [19].

The study aimed to check the effectiveness of removing selected pesticides (malathion, fenitrothion, and p,p′-DDT) from the surfaces of lemons and vegetables using commercial kitchen EWDs. To the best of our knowledge, the removal of pesticides from vegetables and fruits using kitchen EWDs remains largely unknown. The effectiveness of EWDs was additionally compared with traditional methods: washing with tap water, washing with water and detergent, and washing with hot water.

## 2. Results and Discussion

**Figure 1:**
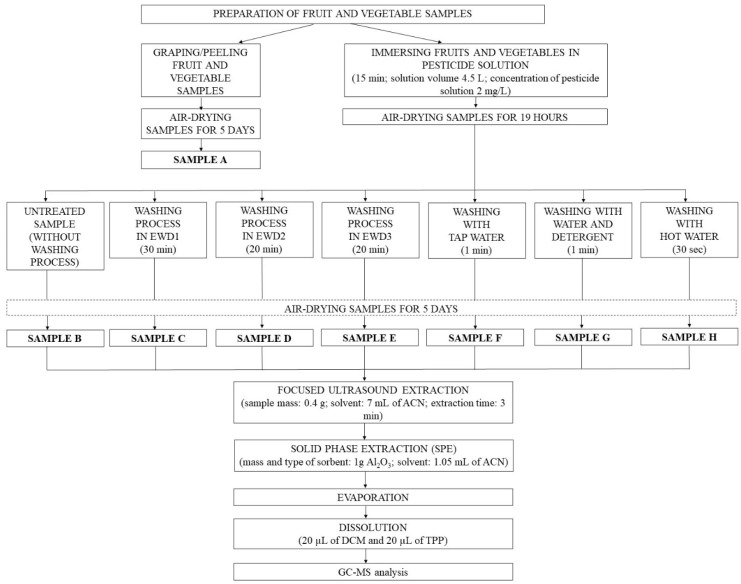
Scheme of the whole procedure of the experiments.

**Figure 2:**
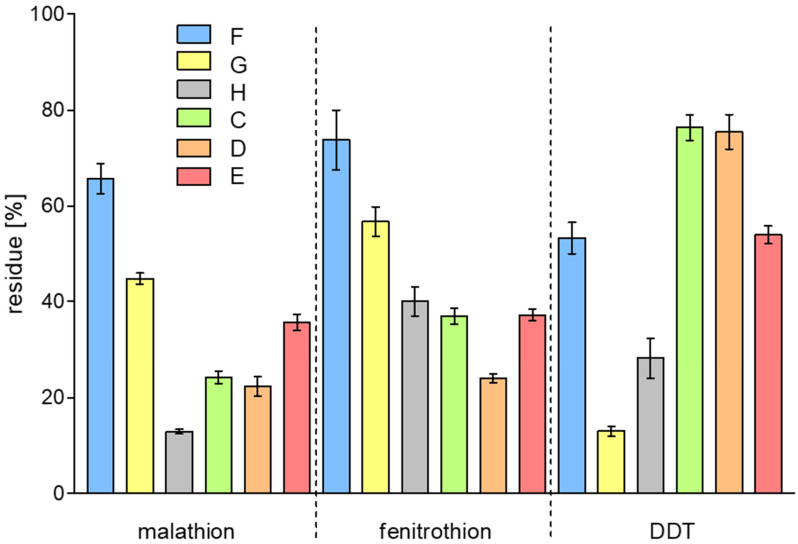
Pesticide residues in lemon after different treatment methods: samples treated in/with F—tap water; G—water and detergent; H—hot water; C—EWD1; D—EWD2; E—EWD3; dashed lines to separate results for individual pesticides. Error bars represented by SEM: if their ranges overlap, there are no significant differences between the compared values.

As a result of the experiments carried out for lemon and selected vegetables (previously soaked in an aqueous solution of pesticides—sample B; using treatment methods with EWDs (samples C–E) and with water (samples F–H), many samples were obtained that were subjected to GC/MS analysis (Figure 1). Based on the chromatograms, the residue % was calculated by dividing the average analyte signal for samples (samples C–H), i.e., after the clean-up method applied by the average analyte signal for the untreated sample (sample B in Figure 1). After the EWDs treatment, the content of malathion and fenitrothion decreased in the lemon peel to approximately 20–40% of the initial concentration (Figure 2). These pesticides have relatively good solubility in water (Table S1). The agents used in EWD kitchen cleaners (electrolyzed water, ultrasonic waves, and UV light) promote the removal of these compounds from the lemon surface. This is additionally facilitated by their degradation, as described in previous studies on pesticide degradation in water. Moreover, the LogP for malathion and fenitrothion is approximately 3 (Table S1), which does not indicate strong sorption of these compounds onto the fruit surface. Traditional methods of washing lemons (tap water—sample F; water and detergent—sample G; hot water—sample H) are also effective, although to varying degrees, in removing pesticides (Figure 2) from the surface of the fruit. An increase in the efficiency of the washing process can be observed in the case of additional agents, i.e., detergent or elevated temperature.

**Figure 3:**
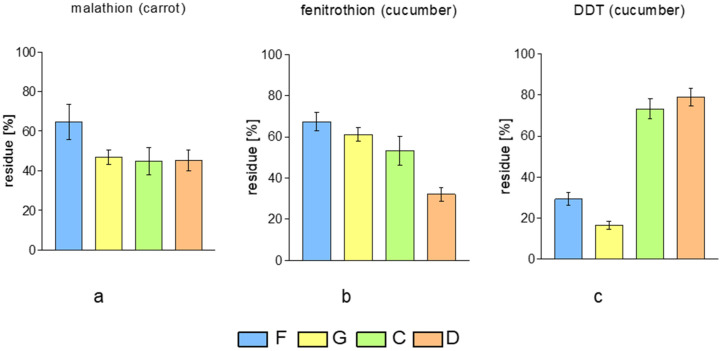
Pesticide residues in vegetables after different treatment methods: samples treated in/with F—tap water; G—water and detergent; C—EWD1; D—EWD2: (**a**) malathion, (**b**) fenitrothion, (**c**) DDT. Error bars represented by SEM: if their ranges overlap, there are no significant differences between the compared values.

In the case of experiments conducted for carrots, the use of EWDs caused a reduction in the malathion content by more than half (Figure 3a). The highest fenitrothion removal efficiency in the case of cucumber was obtained for sample D (reduction above 60%)—Figure 3b. For comparison with classical methods, in both cases, the lowest washing efficiency was obtained for the method using tap water (sample E); although, in this case, the original concentration of pesticides decreased by about 30% (Figure 3a,b). In the case of DDT, a compound with low solubility in water, traditional methods turned out to be more effective compared to EWDs treatment, for which its removal efficiency is from 20 to 40% (Figure 3). This is probably a result of the partially mechanical removal of the wax layer [20] from the lemon surface during washing by hand (rubbing). Additionally, an increase in the temperature (hot water—sample H) during washing and interactions with surfactants promotes the removal of the wax layer. Due to the high LogP for DDT, it can be expected that this pesticide is strongly accumulated in the waxes on the surface of the lemon. The use of EWDs (samples C and D) to wash cucumber to remove DDT (Figure 3c) gives similar results as in the case of lemon (Figure 1), a concentration reduction of about 20%. Based on our studies, it was found that the effectiveness of EWDs in removing pesticides from lemons and vegetables surface ranged from 20 to 80%. In a similar study, Calvo et al. showed that electrolyzed water caused a pesticide reduction not exceeding 40% (cyprodinil, tebuconazole, and iprodione from the surface of peaches, nectarines, and apricots) [21]. EWDs show a promising reduction in pesticides from lemons and vegetables.

The results of our study on the purification of lemon and vegetables using traditional methods are similar to the results of the literature presented in Table S2 [1,15,16,17,20,22,23,24,25,26,27,28,29,30,31,32,33,34,35,36,37,38,39,40,41,42,43,44,45,46]. Other researchers have also found that washing with tap water is insufficient for removing residual concentrations of pesticides. Manually washing vegetables and fruits containing different pesticides in tap water results in different efficacies depending on their solubility in water and affinity to organic matter, usually expressed by LogP [44]. Frequent rubbing during washing and rinsing for a long time in running water can increase the degree of pesticide reduction [20,47]. The next method used by the authors of the manuscript was treating lemon and vegetables with detergent and rinsing with water. The results of pesticide reduction were at the level of 25–75%. Among the tested compounds, DDT was the most removable, which was washable from over 50% from cucumber and 75% from lemon. Other authors also obtained similar degrees of pesticide removal using detergent and water washing, which were at the level of 30–85%. (Table S2). Mir et al. found that the detergent solution improved the efficiency of pesticide removal, but the disadvantage of this process was that it negatively affected the nutritional and organoleptic value of vegetables [48]. The next process tested by the authors was treatment with boiling water at a temperature of 100 °C. The authors used lemon for the study. Using this method, a 50–90% reduction in pesticides was achieved. The best elimination results were achieved when the tested compound was malathion. The results obtained are to some extent consistent with those obtained by Kruve A et al. for thiabendazole, which is characterized by properties similar to malathion and fenitrothion (LogP = 2.47, WS = 50 mg L^−1^) [49]. In the mentioned work, the effect of different orange washing methods on the content of this pesticide in the fruit peel was assessed. The least effective method was washing with cold water, while the most effective was washing with hot water. The literature data show that other food products such as strawberries, cabbage, perilla leaves, ssamchoo, and spinach were subjected to the hot water washing process (pesticide reduction 0–93%) (Table S2). The efficiency of contaminant reduction depends on the time and temperature during the procedure. However, the disadvantage of this process is that degradation products of pesticide residues may be formed, which are also toxic, which increases the risk to human health [13,50].

To increase the effectiveness of cleaning vegetables and fruits from pesticides, scientists add various chemical substances: sodium bicarbonate, chlorine, alkaline water, vinegar, and ozone [45]. It is worth noting that the use of bicarbonate in the case of eliminating thiabendazole and phosmet was effective and reduced the level of contamination by up to 80–96% [16]. The application of ozonation also seems to be a promising method of pesticide removal. Rodrigues et al., studying the elimination of azoxystrobin, chlorothalonil, and difenoconazole from tomatoes, showed a reduction of 70–90% [46]. To achieve better pesticide removal efficiency from food, radiation (reduction of 4–97%), cold plasma (reduction of 45–99%), and ultrasound (reduction of 16–92%) are also used (Table S2). Using chemicals and advanced purification technologies improves the treatment properties of water. Still, in many cases, it can also lead to the formation of transformation products that may be more toxic than the parent compounds [48].

## 3. Methodology

### 3.1. Chemical Reagents

Malathion, p,p′-DDT, and fenitrothion standards were purchased from Łukasiewicz Research Network—Institute of Industrial Organic Chemistry (Warsaw, Poland); triphenyl phosphate (TPH), aluminum oxide, and n-nonane were obtained from Sigma-Aldrich (St. Louis, MO, USA). Acetonitrile and dichloromethane were purchased from Honeywell (Seelze, Germany). All reagents were of chromatographic grade. Standard stock solutions (100 mg mL^−1^) of each pesticide were prepared in acetonitrile and stored in a sealed flask at 4 °C. A pesticide mixture (2 mg L^−1^) was prepared by diluting the standard stock solution with water.

### 3.2. Electrolyzed Water Devices

In the study, EWDs were used to remove pesticides from the surfaces of vegetables and fruits. The EWDs used in the study are kitchen appliances available on the market that are intended for cleaning food (vegetables, fruits, meat, and fish). According to the manufacturer’s description, EWDs can also be used to remove pesticide residues. The devices differed in shape, efficiency, work programs, and additional functions. A program dedicated to fruits and vegetables was selected for each tested device. Table 1 presents the specification of the EWDs used during the tests, and Table S3 presents the detailed technical data of the devices.

### 3.3. Preparation of Samples

The cucumber, carrot, and lemon samples were randomly purchased from a local grocery store (Bydgoszcz, Poland) and kept at 4 °C until analysis. Samples (fruit and vegetable peels) were pre-analyzed and determined to be free of previous residues of pesticides before the experiment (sample A). The lemons and vegetables were soaked in 4.5 L of mixed pesticide solution (concentration 2 mg L^−1^) for 15 min to ensure correct application of pesticides. Contaminated samples were air-dried for 19 h at room temperature. The dried fruit and vegetable peels untreated by the washing process were control samples (sample B). Subsequently, the rest of the contaminated vegetables and fruits were washed using six methods (Table S4): washing in EWDs (sample C–E), running with tap water (sample F), washing with detergent and water (sample G), and washing with hot water (sample H) Then, all samples were air-dried for 5 days. Each process of washing was repeated five times.

### 3.4. Focused Ultrasound Extraction

An ultrasonic processor—Hielscher USA Inc. (Ringwood, NJ, USA), model UP100H (100 W, 30 kHz)—was used with a 5 mm diameter probe. The 0.4 g sample of vegetables and fruits was mixed with 7 mL of ACN in an 8 mL vial and was placed in the ultrasonic chamber. Ultrasonication was carried out at 100% amplitude and room temperature. Treatment time was 3 min. The collected extracts were subjected to the solid phase extraction (SPE).

### 3.5. Solid Phase Extraction

The purification of the analytes was performed using the 6 mL cartridges (Supelco, Bellefonte, PA, USA) with 1 g of Al_2_O_3_ as a sorbent. The cartridge was conditioned with 1.5 mL ACN. Next, an aliquot of a 7 mL sample and two samples of 0.4 mL of the ACN used to wash the sample vials were pumped through the cartridge. Then, the sorbent was washed with 0.25 mL of ACN. The 100 μL of n-nonane (as a keeper solvent) was added to the extract. The eluate was evaporated to about 100 µL under a gentle stream of nitrogen at 40 °C. The residue was redissolved in 200 μL DCM and 20 μL TPH (internal standard: conc. 60 μg mL^−1^ in DCM). The sample was mixed and subjected to GC/MS analysis. The scheme of the whole procedure of preparation is presented in Figure 3.

### 3.6. GC/MS Analysis and Data Processing

The obtained extracts were analyzed by GC/MS in SIM mode (7890B gas chromatograph and the 5977B mass spectrometer (Agilent, Santa Clara, CA, USA). An extract volume of 3 µL was injected in pressure pulsed splitless mode (290 °C): initial pressure 0.2 MPa (30 p.s.i.) for 1.3 min, decreased to constant flow of carrier gas (helium @ 1.5 mL min^−1^). Chromatographic capillary column, ZB-5MS (30 m × 0.25 mm × 0.25 μm) was used for the separation. The analysis was performed using the following oven temperature program: 50 °C (1.5 min), then 35 °C/min to 180 °C and 20 °C/min to 280 °C (19.8 min). The samples were analyzed by GC/MS in both SCAN (mass to charge ratio in the range of 50–450 amu) and SIM mass spectrometer modes (monitored ions: Table S4). Chromatograms in SCAN mode were used to confirm the correct identification of the substance by comparing the obtained spectrum with the library spectrum (from the NIST17 spectrum library—NIST, Gaithersburg, MD, USA). GC/MS (SIM) chromatograms were used for quantitative analysis and to confirm the correctness of chromatogram separation (Q1, Q2 ions—Table S5).

All the chromatograms were analyzed using MassHunter Quantitative Analysis (ver. B.08.00 (Agilent, Santa Clara, CA, USA). The obtained results were then processed with the RKWard software ver. 0.7 (R Foundation for Statistical Computing, Vienna, Austria); for the outlier tests, MS Excel 365 (Microsoft Co., Redmont, WA, USA); for recalculations. KyPlot software ver. 6.0.2 (KyensLab Incorporated., Tokyo, Japan) was used for the chart creation and for the ANOVA analysis with Tukey’s test (differences were considered significant at *p* < 0.05).

## 4. Conclusions

Pesticides can cause a number of severe health effects and illnesses, even at trace concentrations. Therefore, the levels of pesticide residue that remain in the fruits and vegetables both pre- and post-harvest could be effectively reduced by various household methods. However, conventional methods such as washing with water, water and detergent, hot water, and the use of chemical compounds have several disadvantages such as low pesticide removal efficiency, the deterioration of the organoleptic properties of food, and undesirable effects related to the formation of transformation products. Therefore, scientists are improving traditional methods for removing pesticides from food products and also using advanced technologies such as irradiation, electrolysis, pulsed electric field, ultrasound, etc. Due to the different species of vegetables, fruits, and cereals used for testing, as well as different pesticides, it is difficult to compare the results and draw conclusions. In addition, most studies are conducted on a laboratory scale; hence, there is a need to translate scientific and laboratory research into commercial utility. In our studies, we also used commercially available EWDs, and, to our knowledge, this is the first study of this type conducted using kitchen appliances. Tested EWDs enable the effective removal of the selected pesticide residues from the surface of lemons and selected vegetables. The degree of pesticide removal depends on their properties—those that are more soluble in water (such as malathion and fenitrothion) are removed to a greater extent than DDT with strong lipophilic properties (reduction from 20 to 40%). The best effectiveness of EWDs was observed in the case of malathion removal; in the case of lemon, on average for the three tested devices, this was about 70%, and in the case of carrot, this was almost 60%. The highest degree of reduction in fenitrothion and malathion concentration was obtained for EWD2, for which the reduction in concentration was up to 20–30% of the initial concentration, while the highest effectiveness in DDT removal was obtained in EWD3. The variable effectiveness of traditional washing methods was also applied, using agents such as tap water, water with detergent, and hot water. In some cases, the methods mentioned were found to be comparable or better (e.g., removal of fenitrothion from lemon) than those using EWDs. Tap water washing usually gave the smallest reduction in concentration, although noticeable (statistically significantly different results compared to the original concentration). The undoubted advantage of using EWDs is the lack of need for an operator during the processing techniques, which may be particularly important when preparing larger portions of fruit or vegetables. However, extensive further research about removing other pesticides in EWDs for a wide range of fruits and vegetables needs to be conducted. The further development and optimization of techniques for reducing residual concentrations of pesticides in vegetables and fruits is necessary, especially for commercial use.

## Figures and Tables

**Table 1:** The specification of the EWDs.

	EWD1	EWD2	EWD3
Rated Power	90 W	72 W	85 W
Time of the electrolyzed water generation	30 min	20 min	20 min
UV LED wavelength	275 nm	275 nm	275 nm
Capacity	12 L	9 L	9 L

## Data Availability

The data presented in this study are available on request from the corresponding author.

## Supplementary Materials

The following supporting information can be downloaded at https://www.mdpi.com/article/10.3390/molecules29235797/s1, Table S1. Properties of pesticides; Table S2. Comparison of methods for removing residual pesticide concentrations in food products; Table S3. Technical data of the EWDs; Table S4. Description of individual treatment methods. Table S5. Characteristic fragment ions of analyte mass spectra.
