# Supplementary material for: Removal of Pesticides from Lemon and Vegetables Using Electrolyzed Water Kitchen Devices
Source: Molecules. 2024 Dec 8;29(23):5797. doi: 10.3390/molecules29235797 (PMC11643934; doi:10.3390/molecules29235797)
Supplement: Supplementary file 1 [file molecules-29-05797-s001.zip › molecules-3288827-supplementary.pdf]

Removal of Pesticides from Lemon and Vegetables Using Electrolyzed Water Kitchen Devices

Waldemar Studziński *, Izabela Narloch * and Łukasz Dąbrowski

Department of Food Analysis and Environmental Protection, Faculty of Chemical Technology and Engineering, Bydgoszcz University of Science and Technology, Seminaryjna 3, 85-326 Bydgoszcz, Poland; lukas@pbs.edu.pl

* Correspondence: waldemar.studzinski@pbs.edu.pl (W.S.); izabela.narloch@pbs.edu.pl (I.N.); Tel.: +48-52-374-90-67 (I.N.)

Table S1. Properties of pesticides

	**BP** [°C]	**VP** [mmHg]	**WS** [mg/L]	**LogP**	**Chemical structure**
**malathion**	351[e]	$3.38 \cdot 10^{-6}$	143	2.36	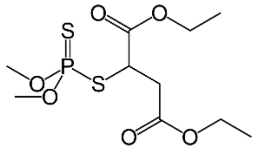
**fenitrothion**	360[e]	$5.40 \cdot 10^{-5}$	38	3.3	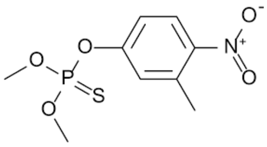
**DDT**	260	$1.60 \cdot 10^{-7}$	0.0055	6.91	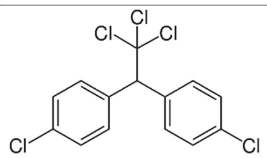

[e] – evaluated value, BP – boiling point, VP – vapor pressure, WS – water solubility

Table S2. Comparison of methods for removing residual pesticide concentrations in food products

**Food material**	**Type of pesticide**	**Processing conditions**	**Percentage reduction**	**Reference**
		Tap water		
Strawberry	Acetamiprid, Alpha-cypermethrin, Boscalid, Bupirimate, Chlorpyrifod, Cyprodinil, Deltamethrin, Fenhexamid, Fludioxonil, Folpet, Iprodione, Lambda-cyhalothrin, Pirimicarb, Pyraclostrobin, Tetraconazole, Trifloxystrobin	Treatment time 1-5 min	2-68	[1]
Cucumber	Trichlorfon, Dimethoate, Dichlorvos, Fenitrothion, Chlorpyrifos	Treatment time 5-20 min	13-63	[2]
Lettuce	Diniconazole, Chlorfenapyr, Imidacloprid, Thiamethoxam,	Running water for 5 min	50-90	[3]
		Stagnant water for 5 min	45-65	

	Chlorantraniliprole, Fludioxonil, Lufenuron, Indoxacarb, Azoxystrobin, Pyraclostrobin			
Perilla leaves	Diniconazole, Chlorfenapyr, Imidacloprid, Thiamethoxam, Chlorantraniliprole, Fludioxonil, Lufenuron, Indoxacarb, Azoxystrobin, Pyraclostrobin	Running water for 5 min<br>Stagnant water for 5 min	40-90<br>40-80	[3]
Spinach	Diniconazole, Chlorfenapyr, Imidacloprid, Thiamethoxam, Chlorantraniliprole, Fludioxonil, Lufenuron, Indoxacarb, Azoxystrobin, Pyraclostrobin	Running water for 5 min<br>Stagnant water for 5 min	70-95<br>40-55	[3]
Ssamchoo	Diniconazole, Chlorfenapyr, Imidacloprid, Thiamethoxam, Chlorantraniliprole, Fludioxonil, Lufenuron, Indoxacarb, Azoxystrobin, Pyraclostrobin	Running water for 5 min<br>Stagnant water for 5 min	40-80<br>20-50	[3]
Black & green oilve	Chlorypirfos, Cypermethrin, Cyhaltorin	Running water for 1 min	26-48	[4]
Potatoes	DDT	Running water for 1 min	18	[4]
Lemons	Imazilil	Running water for 1 min	42	[4]
Orange	Abamectin, Buprofezin, Imazalil, Thiophanate-Methyl, Etoxazole	Stagnant water for 20 min	3-68	[5]
Orange	5 pesticides	Running water for 1-2 min	26-84	[6]
		Water with additional reagent		
Apple	Thiabendazole, Phosmet	Sodium bicarbonate solution (10 mg/ml), treatment time 12 and 15 min	80-96	[7]
Lettuce	Diniconazole, Chlorfenapyr, Imidacloprid, Thiamethoxam, Chlorantraniliprole, Fludioxonil, Lufenuron, Indoxacarb, Azoxystrobin, Pyraclostrobin	Water with detergent for 5 min<br>2% sodium bicarbonate solution for 5 min<br>Alkaline water for 5 min<br>5% vinegar for 5 min	50-80<br>50-85<br>60-85<br>40-70	[3]
Perilla leaves	Diniconazole, Chlorfenapyr, Imidacloprid, Thiamethoxam, Chlorantraniliprole, Fludioxonil, Lufenuron, Indoxacarb, Azoxystrobin, Pyraclostrobin	Water with detergent for 5 min<br>2% sodium bicarbonate solution for 5 min<br>Alkaline water for 5 min	30-70<br>40-70<br>40-90	[3]

		5% vinegar for 5 min	30-70	
Spinach	Diniconazole, Chlorfenapyr, Imidacloprid, Thiamethoxam, Chlorantraniliprole, Fludioxonil, Lufenuron, Indoxacarb, Azoxystrobin, Pyraclostrobin	Water with detergent for 5 min	30-60	[3]
		2% sodium bicarbonate solution for 5 min	40-70	
		Alkaline water for 5 min	40-90	
		5% vinegar for 5 min	30-80	
Ssamchoo	Diniconazole, Chlorfenapyr, Imidacloprid, Thiamethoxam, Chlorantraniliprole, Fludioxonil, Lufenuron, Indoxacarb, Azoxystrobin, Pyraclostrobin	Water with detergent for 5 min	10-50	[3]
		2% sodium bicarbonate solution for 5 min	0-40	
		Alkaline water for 5 min	20-50	
		5% vinegar for 5 min	10-40	
Orange	Abamectin, Buprofezin, Imazalil, Thiophanate-Methyl, Etoxazole	Stagnant water with 10% Sodium carbonate for 20 min	38-83	[5]
		Stagnant water with 8% Acetic acid for 20 min	18-81	
		Stagnant 100% Apple vinegar for 20 min	17-88	
		Boiling water		
Strawberry	Acetamiprid, Alpha-cypermethrin, Boscalid, Bupirimate, Chlorpyrifod, Cyprodinil, Deltamethrin, Fenhexamid, Fludioxonil, Folpet, Iprodione, Lambda-cyhalothrin, Pirimicarb, Pyraclostrobin, Tetraconazole, Trifloxystrobin	Boiling water (100 °C) for 1, 2 and 5 min	11-93	[1]
Letuce	Diniconazole, Chlorfenapyr, Imidacloprid, Thiamethoxam, Chlorantraniliprole, Fludioxonil, Lufenuron, Indoxacarb, Azoxystrobin, Pyraclostrobin	Boiling water (100 °C) for 5 min	30-90	[3]
Perilla leaves	Diniconazole, Chlorfenapyr, Imidacloprid, Thiamethoxam, Chlorantraniliprole, Fludioxonil, Lufenuron,	Boiling water (100 °C) for 5 min	20-90	[3]

	Indoxacarb, Azoxystrobin, Pyraclostrobin			
Spinach	Diniconazole, Chlorfenapyr, Imidacloprid, Thiamethoxam, Chlorantraniliprole, Fludioxonil, Lufenuron, Indoxacarb, Azoxystrobin, Pyraclostrobin	Boiling water (100 °C) for 5 min	40-90	[3]
Ssamchoo	Diniconazole, Chlorfenapyr, Imidacloprid, Thiamethoxam, Chlorantraniliprole, Fludioxonil, Lufenuron, Indoxacarb, Azoxystrobin, Pyraclostrobin	Boiling water (100 °C) for 5 min	0-90	[3]
		Cold plasma		
Tomato	Chlorpyrifos	5 W for 6 min	89	[8]
Mango	Chlorpyrifos, Cypermetrin	8 kV for 5 min	63-74	[9]
Lycium barbarum	Dichlorovos, Omethoate	10 kV for 30 min	96.8-99.5	[10]
Blueberry	Boscalid, Imidacloprid	80 kV for 5 min	75-80	[11]
Apple	Chlorpyrifos, Diazinon	13 kV for 10 min	58-87	[12]
Apple	Paraoxon	4 kV for 2 min	96	[13]
Cucumber	Chlorpyrifos, Diazinon	13 kV for 10 min	58-82	[12]
Cucumber	Diazinon	0.75 W for 15 min	88	[14]
Strawberry	Azoxystrobin, Cyprodinil, Fludioxonil, Pyriproxyfen	80 kV for 5 min	45-71	[15]
Soybean	Chlorpyrifos	6 min	70	[16]
		Irradiation		
Strawberry	Azoxystrobin, Carbendazim	4 kGy	28-56	[17]
Pea	Profenosfos	32 kGy	48	[18]
Cucumber, tomato, capsicum	Chlorpyrifos, Diazinon, Phosphamidon	1 kGy	80-95	[19]
Potateos	Pirmiphos-methyl, Malathion	1 kGy	18	[20]
Grapes	Pirmiphos-methyl, Cypermethrin	7 kGy	4-19	[20]
Dates	Pirmiphos-methyl	7 kGy<br>1 kGy	3-44	[20]
dried peppers	Carbendazim, Dimethomorph, Fluquinconazol, Imidacloprid, Myclobutanil, Tetraconazole, Thiamethoxam	UV254<br>UV360<br>UV254 + ozone	up to 60<br>up to 13<br>up to 97	[21]
		Sonication		

Tomato	Captan, Metalaxyl, Thiamethoxam	Three different currents (200, 800 and 1400mA) of 10 min treatment. Two kinds of US treatments including ultrasonic bath (UB) at 40kHz and ultrasonic probe (UP) at 24 kHz were tested	82-93	[22]
Strawberry	Acetamiprid, Alpha-cypermethrin, Boscalid, Bupirimate, Chlorpyrifod, Cyprodinil, Deltamethrin, Fenhexamid, Fludioxonil, Folpet, Iprodione, Lambda-cyhalothrin, Pirimicarb, Pyraclostrobin, Tetraconazole, Trifloxystrobin	Ultrasonic cleaner at 40 kHz, power 2×240W peak/period for 1, 2 and 5 min	16-91	[1]
Cucumber	Trichlorfon, Dimethoate, Dichlorvos, Fenitrothion, Chlorpyrifos	Ultrasonic bath at 40kHz for 5-20 min	16-84	[2]
Letuce	Diniconazole, Chlorfenapyr, Imidacloprid, Thiamethoxam, Chlorantraniliprole, Fludioxonil, Lufenuron, Indoxacarb, Azoxystrobin, Pyraclostrobin	Ultrasonic cleaner was maintained at 40 kHz for 5 min	50-80	[3]
Perilla leaves	Diniconazole, Chlorfenapyr, Imidacloprid, Thiamethoxam, Chlorantraniliprole, Fludioxonil, Lufenuron, Indoxacarb, Azoxystrobin, Pyraclostrobin	Ultrasonic cleaner was maintained at 40 kHz for 5 min	40-85	[3]
Spinach	Diniconazole, Chlorfenapyr, Imidacloprid, Thiamethoxam, Chlorantraniliprole, Fludioxonil, Lufenuron, Indoxacarb, Azoxystrobin, Pyraclostrobin	Ultrasonic cleaner was maintained at 40 kHz for 5 min	40-60	[3]
Ssamchoo	Diniconazole, Chlorfenapyr, Imidacloprid, Thiamethoxam, Chlorantraniliprole, Fludioxonil, Lufenuron, Indoxacarb, Azoxystrobin, Pyraclostrobin	Ultrasonic cleaner was maintained at 40 kHz for 5 min	10-80	[3]
Cucumber	Chlorypirfos, Cypermethrin, Cyhaltorin, DDT, Imizalil	Ultrasonic cleaner 300W	49-83	[4]
Perilla leaves	Carbamates, Pyrethroids	Ultrasonic cleaner 240 W for 15 min	up to 60	[23]
		Ozonation		

Tomato	Azoxystrobin, Chlorothalonil, Difenoconazole	3 ppm concentration, 30 min. of treatment time	70-90	[24]
Table grapes	Azoxystrobin, Bupirimate, Carbendazim, Penconazole, Triadimenol, Trifloxystrobin, Chlorpyrifos	Ozone enriched air (0.64 mg m^{-3})	39-98	[25]
Carrot	Difenoconazole, Linuron	10 ppm concentration, 2 h treatment time	95-98	[26]
Maize	Pirimiphosmethyl	Ozone gas at a concentration of 0.86 mg/L continuous flow rate of 1.0 L/min	91	[27]
Apple	Captan, Boscalid, Pyraclostrobin	10 ppm concentration, 30 min of treatment time	42-95	[28]
Wheat	Bifenthrin, Pirimiphosmethyl	180 min ozone exposure	38-71	[29]
Wheat	Deltamethrin, Fenitrothion	180 min of ozone exposure	67-89	[30]
Strawberry	Acetamiprid, Alpha-cypermethrin, Boscalid, Bupirimate, Chlorpyrifod, Cyprodinil, Deltamethrin, Fenhexamid, Fludioxonil, Folpet, Iprodione, Lambda-cyhalothrin, Pirimicarb, Pyraclostrobin, Tetraconazole, Trifloxystrobin	Fruits were immersed in this solution (20 °C, 1 mg O_3/L) for 1, 2 and 5 min	10-75	[1]
Soybean	Chlorpyrifos	550 mg O_3/L for 30 min	up to 50	[16]

Table S3. Technical data of the EWDs.

	**EWD1**	**EWD2**	**EWD3**
Voltage/Frequence	240V/50Hz	220V/50Hz	220V/50Hz
Rated Power	90 W	72 W	85 W
Stanby Power	<2 W	<2 W	<2 W
Time of the electrolyzed water generation	up to 30 min	up to 20 min	up to 20 min
Electrolytic Modes	Fruits and vegetables: 15-30 min<br>Fish: 18-30 min	Meat: 15-20 min<br>Fruits and vegetables: 12-20 min	Meat: 11-20 min<br>Fruits and vegetables: 11-20 min

	Baby items: 30 min	Seafood: 20 min<br>Utensil: 8-20 min<br>Disinfectant Key: 20 min	Seafood: 6-20 min<br>Tableware: 6-20 min<br>Disinfectant Key: 20 min
Ultrasonic Working Time	-	-	Meat: 8 min<br>Fruits and vegetables: 5 min<br>Seafoof: 10 min<br>Tableware: 7 min<br>Does not work in disinfection mode
UV LED wavelength	275 nm	275 nm	275 nm
Product Size	279x279x353 mm	447x275x318 mm	447x275x318 mm
Capacity	12 L	9 L	9 L
Net weight	3.2 kg	4.85kg	5.5kg

Table S4. Description of individual treatment methods

Series Number	Description
Sample A	blank test - lemons, and vegetables not contaminated with pesticides
Sample B	lemons and vegetables contaminated with pesticides
Sample C	lemons and vegetables contaminated with pesticides and treated in EWD1 for 30 minutes
Smaple D	lemons and vegetables contaminated with pesticides and treated in EWD2 for 20 minutes
Sample E	lemons and vegetables contaminated with pesticides and treated in EWD3 for 20 minutes
Sample F	lemons and vegetables contaminated with pesticides and treated with tap water for 1 minute
Sample G	lemons and vegetables contaminated with pesticides and cleaned with detergent for 1 min and rinsed with tap water
Sample H	lemons contaminated with pesticides and cleaned with hot water (100ºC) for 1 minute

Table S5. Characteristic fragment ions of analyte mass spectra

	Tgt	Q1	Q2	$[M]^+$	MS Signals*, m/z Characteristic Fragment Ions
**fenitrothion**	277	260	125	277	277, 125, 109, 260, 79
**malathion**	173	158	125	330	125, 173, 93, 127, 158
**DDT**	235	237	165	352	235, 237, 165, 236, 199

*Base peak ion underlined, Tgt – target ion, Q1, Q2 – qualifier ions, M^+ – molecular ion
